# HDAC6 Triggers the ATM-Dependent DNA Damage Response To Promote PRV Replication

**DOI:** 10.1128/spectrum.02132-22

**Published:** 2023-03-23

**Authors:** Weiyin Xu, Ping Yan, Ziyan Zhou, Jingting Yao, Haochun Pan, Luyao Jiang, Zongyi Bo, Bo Ni, Mingxia Sun, Song Gao, Changchao Huan

**Affiliations:** a Institute of Agricultural Science and Technology Development, College of Veterinary Medicine, Yangzhou University, Yangzhou, China; b Jiangsu Co-Innovation Center for Prevention and Control of Important Animal Infectious Diseases and Zoonoses, Yangzhou, China; c Key Laboratory of Avian Bioproduct Development, Ministry of Agriculture and Rural Affairs, Yangzhou, China; d Institutes of Agricultural Science and Technology Development, Yangzhou University, Yangzhou, China; e China Animal Health and Epidemiology Center, Qingdao, China; f Key Laboratory of Veterinary Biotechnology, Harbin Veterinary Research Institute, Chinese Academy of Agricultural Sciences, Harbin, China; University of Florida

**Keywords:** PRV, HDAC6, DNA damage, p-H2AX, ATM

## Abstract

Pseudorabies virus (PRV) infection is modulated by various cellular host factors. In this study, we investigated the role of histone deacetylase 6 (HDAC6) in this process. We determined HDAC6 expression *in vitro* and performed gene knockout, pharmacological inhibition analyses, immunofluorescence assays, and statistical analyses. We found that the pharmacological and genetic inhibition of HDAC6 significantly decreased PRV replication, whereas its overexpression promoted PRV replication. Additionally, we demonstrated that PRV infection can induce the phosphorylation of histone H2AX and lead to DNA damage response (DDR), and the ataxia telangiectasia mutated (ATM) inhibitor KU55933 inhibits DDR and PRV infection. Mechanistically, the HDAC6 inhibitor tubacin and *HDAC6* knockout can decrease DDR. The results of this study suggested that HDAC6 may be a crucial factor in PRV-induced ATM-dependent DDR to promote PRV replication.

**IMPORTANCE** Pseudorabies virus (PRV) is a member of the subfamily *Alphaherpesvirinae* of the family *Herpesviridae*. PRV infection in swine can lead to high morbidity and mortality of swine, causing huge economic losses. In particular, PRV variants can cause severe damage to the nervous and respiratory systems of humans, revealing that PRV may be a potential zoonotic pathogen. Vaccines for PRV have been developed that can delay or reduce the epidemic, but they currently cannot eliminate this disease completely. Therefore, studies should investigate new targets for the prevention and control of PRV infection. In this study, we demonstrated that HDAC6 can induce ataxia telangiectasia mutated-dependent DNA damage response to foster PRV replication, indicating that HDAC6 is a therapeutic target for PRV infection.

## INTRODUCTION

Pseudorabies virus (PRV) is an enveloped double-stranded DNA virus belonging to the family *Herpesviridae* (1). Livestock and wild mammals, including pigs, cattle, sheep, goats, cats, dogs, and raccoons, are susceptible to PRV. Among these animals, domestic pigs and wild boars are the main natural reservoir for this virus. Porcine pseudorabies is associated with high morbidity and mortality in piglets of all ages (2–4). PRV-infected younger swine are most severely affected and typically exhibit symptoms related to central nervous system infection, whereas older swine exhibit symptoms related to respiratory disease (5). Presently, no treatment is available for porcine pseudorabies, and although vaccines that have been developed can delay or reduce the epidemic, they cannot eliminate the disease completely (6, 7).

The diversity of DNA virus genomes has led to a plethora of strategies for virus DNA replication. The cellular DNA damage response (DDR) is a complex network of signaling pathways. It safeguards cellular DNA to maintain its genomic integrity, both during replication and when exposed to endogenous damage and exogenous agents (8–10). The activation of one of the phosphatidylinositol 3-kinase-related kinases (PIKKs), namely, ataxia telangiectasia mutated (ATM), ataxia telangiectasia and Rad3 related (ATR), or DNA-dependent protein kinase catalytic subunit (DNA-PKCs), can be used to detect the DNA damage and induce DDR (9, 10). Cellular processes, such as DNA replication, DNA repair, and cell cycle control, require a series of phosphorylation events that can induce DDR.

Histone deacetylase (HDACs) are a group of enzymes which largely share the ability to remove acetyl groups from ε-*N*-acetyl lysine amino acids in histones and other proteins (11). HDACs play an important role in regulating both nuclear and cytoplasmic processes, including transcriptional initiation and elongation, protein stability, and multiprotein complex formation. *HDAC6* was cloned from mice and humans as a mammalian homolog of yeast histone deacetylase 1 (12, 13) and contains DAC1 and DAC2, two deacetylase domains, and a zinc finger-ubiquitin binding domain in the C terminus. DAC2 only has deacetylase activity, whereas DAC1 possesses intrinsic E3 ligase activity both *in vitro* and *in vivo* (14). HDAC6 deacetylates nonhistone substrates, including α-tubulin, heat shock protein 90, and cortactin (15–17). HDAC6 has a crucial role in multiple biological processes, such as immune synapse formation, degradation of misfolded proteins, and cell migration (18). Moreover, HDAC6 can restrict many virus infections, for example, HDAC6 can inhibit influenza A virus replication (19, 20), and HIV-1 fusion and infection can be inhibited by HDAC6-mediated deacetylation of tubulin (21). HDAC6 is a major regulator of the innate response against viral infections. HDAC6 decreases Sendai virus infection by upregulating interferon-β expression (22).

The role of HDAC6 in PRV replication is unclear; therefore, we performed this study to investigate the role of HDAC6 in PRV replication. Our study revealed that HDAC6 promoted PRV replication in PK15 and Vero cells and upregulated DDR. These data indicated that HDAC6 triggers DDR to promote PRV replication.

## RESULTS

### PRV infection altered HDAC6 expression in PK15 and Vero cells.

**FIG 1:**
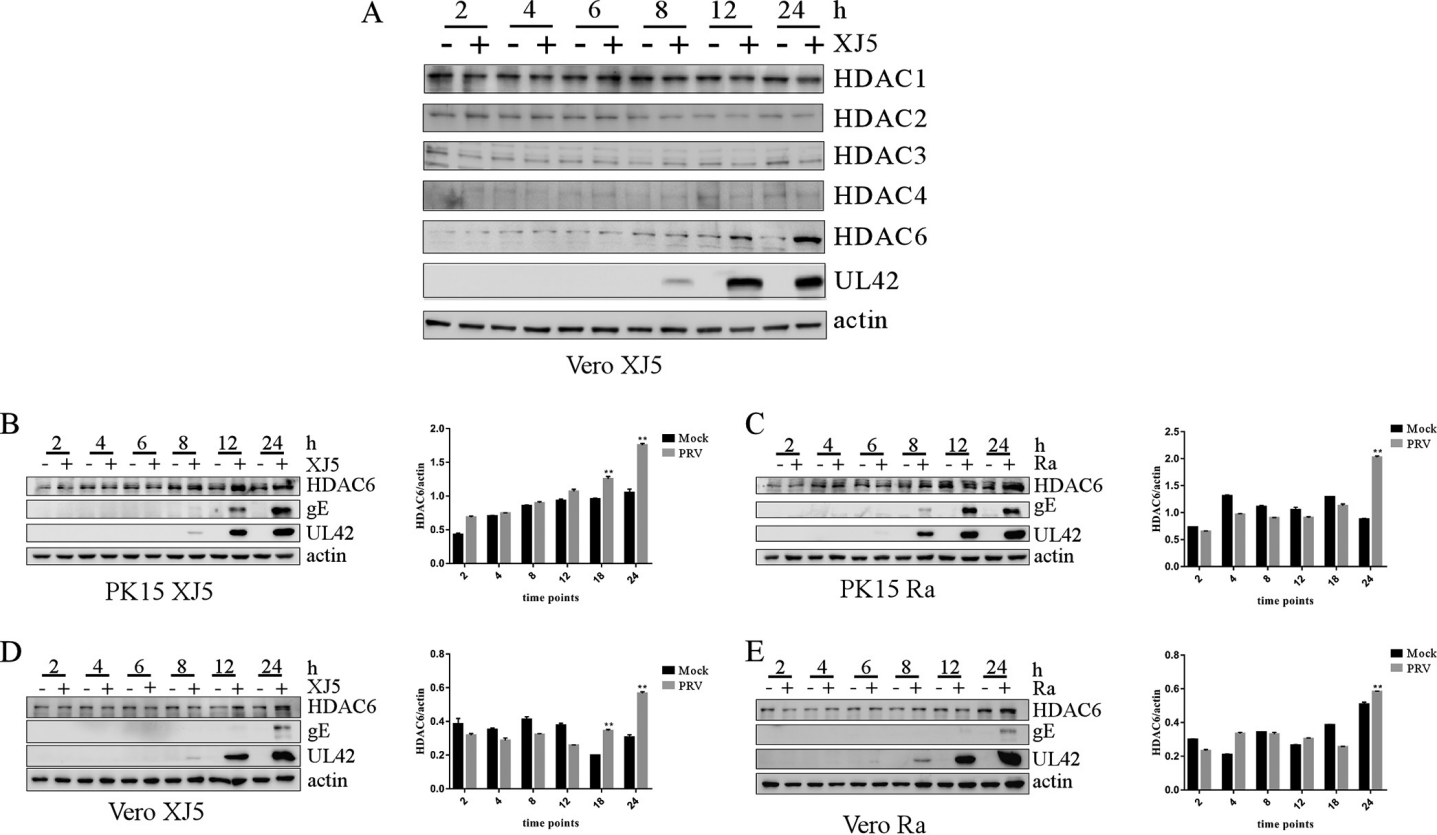
PRV infection induced the expression of HDAC6. (A) PRV XJ5 (MOI, 0.1) was used to infect Vero cells for 2, 4, 8, 12, 18, and 24 h. Western blotting was used to detect HDAC6, PRV UL42, and β-actin proteins in Vero cells. (B to E) PRV XJ5 and Ra (MOI, 0.1) were used to infect PK15 and Vero cells for 2, 4, 8, 12, 18, and 24 h. Western blotting was used to detect HDAC6, PRV UL42, gE, and β-actin proteins in PK15 and Vero cells infected with PRV XJ5 and Ra at different time points. HDAC6-actin visualization relied on Image J. Values represent means ± SD from three independent experiments. **, *P* < 0.01.

HDAC expression in PRV-infected cells was analyzed. Vero cells were infected with PRV XJ5 (multiplicity of infection [MOI] of 0.1). At 2, 4, 6, 8, 12, and 24 h postinfection (hpi), the cells were collected for Western blot analysis. PRV XJ5 infection had no effect on the protein level of HDAC1, HDAC2, HDAC3, or HDAC4, but PRV XJ5 infection increased HDAC6 expression in Vero cells at 12 and 24 hpi (Fig. 1A). Therefore, we focused on the effect of PRV infection on HDAC6 expression. PK15 and Vero cells were infected with PRV XJ5 (MOI, 0.1), respectively. At 2, 4, 6, 8, 12, and 24 hpi, the cells were collected for Western blot analysis. PRV infection increased HDAC6 expression in PK15 and Vero cells at 12 and 24 hpi (Fig. 1B and D). Next, we investigated if PRV Ra infection upregulated HDAC6 expression. Western blot analysis indicated that PRV Ra infection promoted HDAC6 expression in PK15 and Vero cells at 24 hpi (Fig. 1C and E). These results revealed that PRV infection upregulated HDAC6 expression at 24 hpi.

### The HDAC6 inhibitor tubacin decreases PRV replication.

**FIG 2:**
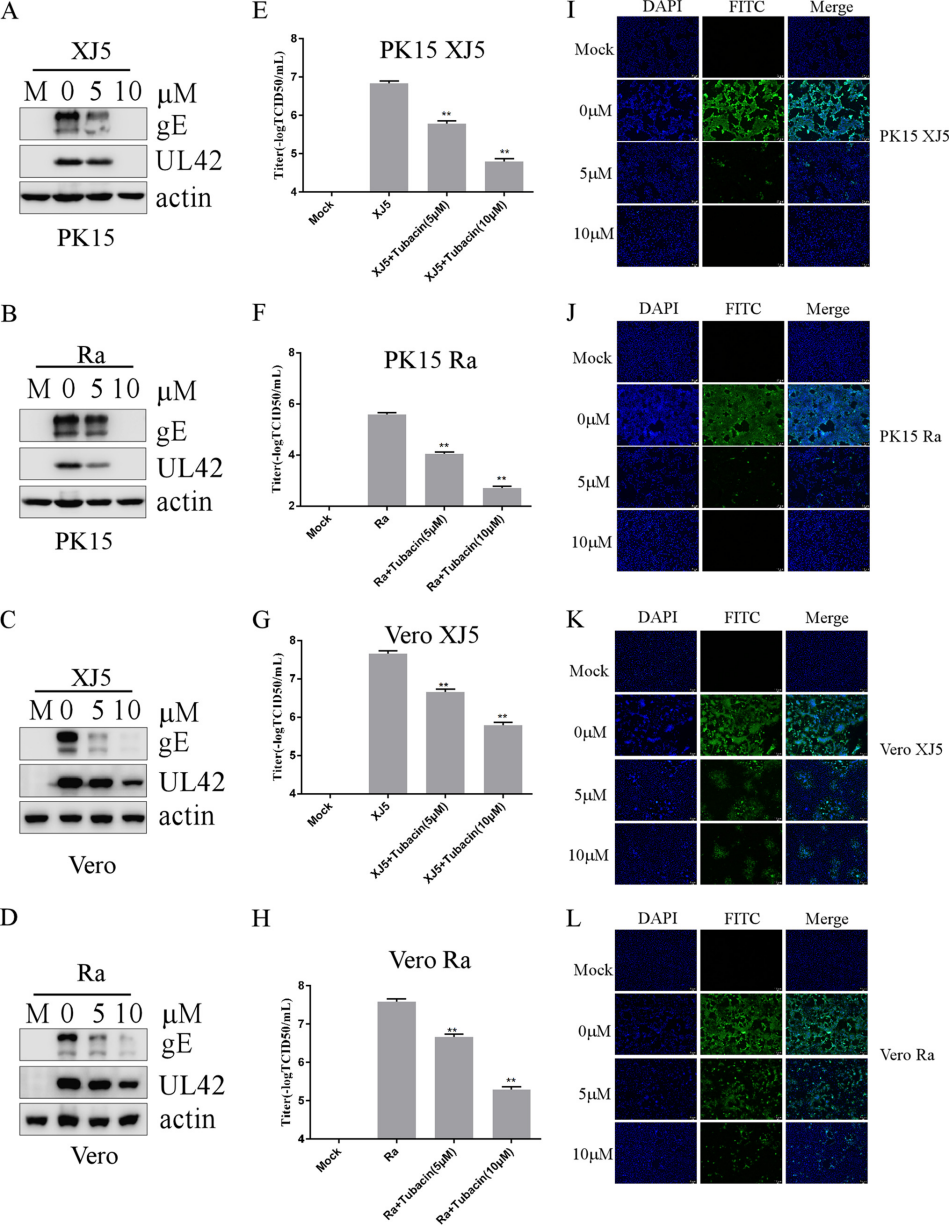
The HDAC6 inhibitor tubacin decreased PRV replication. PK15 and Vero cells were pretreated with different concentrations of tubacin for 1 h before PRV XJ5 or Ra (MOI, 0.1) infection. Then, the cells were treated with different concentrations of tubacin for 24 h. Cells and supernatants were collected at 24 h postincubation. (A to D) PRV gE, UL42, and actin proteins were detected by Western blotting. (E to H) Viral titers were determined using the 50% tissue culture infectious dose assay. (I to L) An immunofluorescence assay was performed to detect PRV infection in cells with PRV-positive serum using probed FITC-conjugated goat anti-pig IgG. Values represent means ± SD from three independent experiments. **, *P* < 0.01.

We first assessed the cytotoxic effect of the HDAC6 inhibitor tubacin for 24 h, and we found no cytotoxic effects of tubacin from 5 to 10 μM (data not shown). The HDAC6 role in PRV replication in PK15 and Vero cells was determined. PK15 and Vero cells were pretreated with HDAC6 inhibitor tubacin (5 and 10 μM) or the control, dimethyl sulfoxide (DMSO) for 1 h at 37°C. Then, the cells were infected with PRV XJ5 (MOI, 0.1) through 24 h of incubation. We found that tubacin downregulated the expression of PRV gE and UL42 proteins (Fig. 2A and C). Tubacin treatment also decreased the number of cells infected with PRV, as determined by immunofluorescence assay (IFA) (Fig. 2I and K). Supernatants were also collected to determine the viral titer, which revealed that tubacin decreased the PRV titer in a dose-dependent manner (Fig. 2E and G). We also observed the effect of tubacin on PRV Ra replication. PRV Ra replication was monitored by Western blotting (Fig. 2B and D), IFA (Fig. 2J and L), and 50% tissue culture infective dose (TCID_50_) assays, which showed that tubacin downregulated gE expression and UL42, the numbers of cells infected with PRV, and PRV titer (Fig. 2F and H). Taken together, these results showed that the HDAC6 inhibitor tubacin decreased PRV replication.

### *HDAC6* knockout decreased PRV replication.

**FIG 3:**
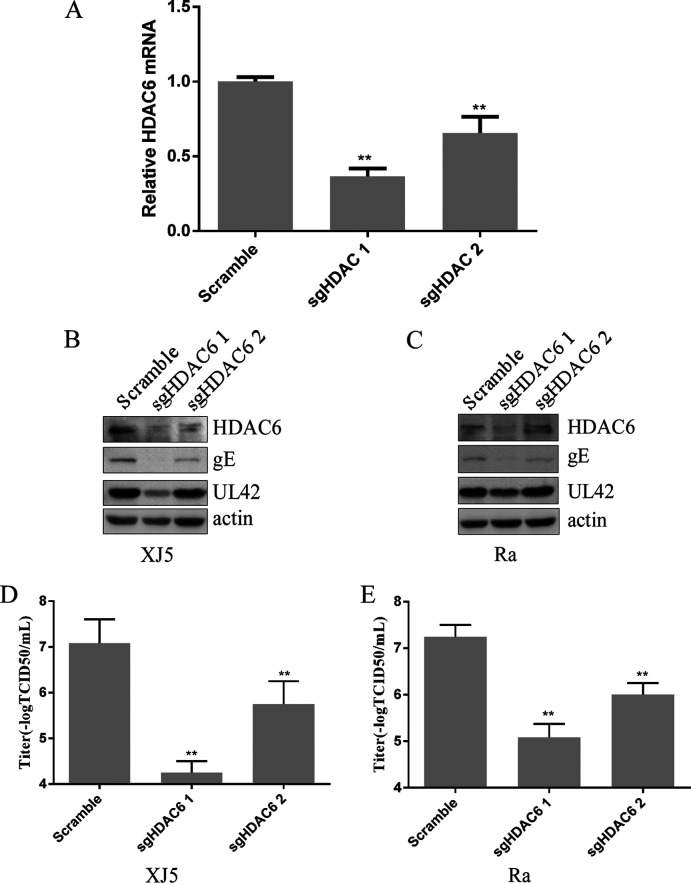
Knockout of *HDAC6* decreased PRV replication. (A) *HDAC6* in PK15 cells was knocked out by the lentivirus packaging system. qPCR was used to detect *HDAC6*. (B to E) PK 15 cells with *HDAC6* knocked out were infected with PRV XJ5 or Ra for 24 h. (B and C) HDAC6, PRV UL42, and gE were measured by Western blotting. (D and E) Viral titers were determined using the 50% tissue culture infectious dose assay. Values represent means ± SD from three independent experiments. **, *P* < 0.01.

To confirm that HDAC6 triggers PRV replication, we knocked out *HDAC6*, downregulating HDAC6 expression in PK15 cells using single guide RNA (sgRNA). Cells were transfected with the negative control (NC) or one of two sgRNAs specifically targeting HDAC6 (sgHDAC6-1 and sgHDAC6-2, respectively). *HDAC6* gene knockout was confirmed by quantitative PCR (qPCR) (Fig. 3A). The cells were infected with PRV XJ5 or Ra for 24 h. Western blot analysis indicated that both sgRNAs against HDAC6 notably downregulated HDAC6 expression (Fig. 3B and C). However, the expression of PRV gE and UL42 was lower in sgHDAC6-1- and sgHDAC6-2-transfected cells than in negative-control (NC)–transfected PK15 cells (Fig. 3B and C). Viral titer indicated that *HDAC6* knockout decreased PRV titer (Fig. 3D and E). These results indicated that *HDAC6* knockout decreased PRV replication.

### HDAC6 facilitates PRV replication.

**FIG 4:**
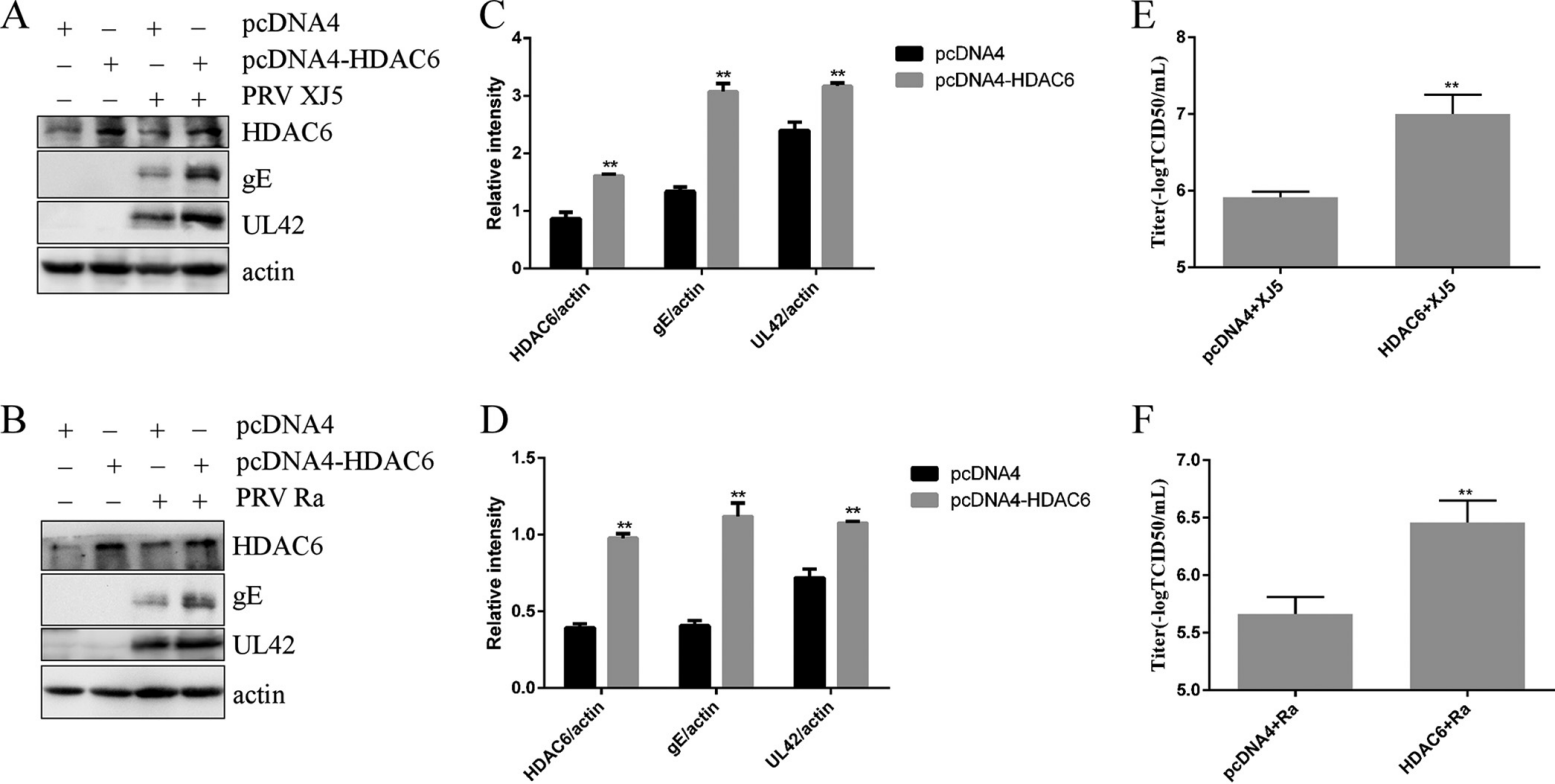
HDAC6 increased PRV replication. HDAC6 was overexpressed in PK15 and Vero cells for 24 h, which were then infected with PRV XJ5 or Ra for 24 h. (A to D) Western blotting was used to detect the levels of HDAC6, PRV UL42, gE, and β-actin proteins. The relative intensity of protein was visualized by Image J. (E and F) Viral titers were determined using the 50% tissue culture infectious dose assay. Values represent means ± SD from three independent experiments. **, *P* < 0.01.

To characterize the role of HDAC6 in PRV infection, we investigated the relationship between HDAC6 expression and PRV replication in Vero cells. We determined if overexpression of HDAC6 in Vero cells increased PRV replication. These cells were transfected with pcDNA4 vector expressing *HDAC6*, and the empty pcDNA4 vector served as the NC. After 24 h, the cells were infected with PRV XJ5 and Ra (MOI, 0.1). After 24 hpi, the cells and supernatants were harvested for further analysis. The Western blot results confirmed that HDAC6 was overexpressed in Vero cells. PRV gE and UL42 expression levels in *HDAC6*-overexpressing Vero cells were increased compared with those in vector control-treated cells (Fig. 4A to D). The effect of HDAC6 expression on the PRV yield was determined by TCID_50_ assay. The data showed higher viral yields in *HDAC6*-overexpressing cells than in vector control-treated Vero cells (Fig. 4E and F). These results indicated that HDAC6 upregulated PRV replication.

### PRV replication induces DDR.

**FIG 5:**
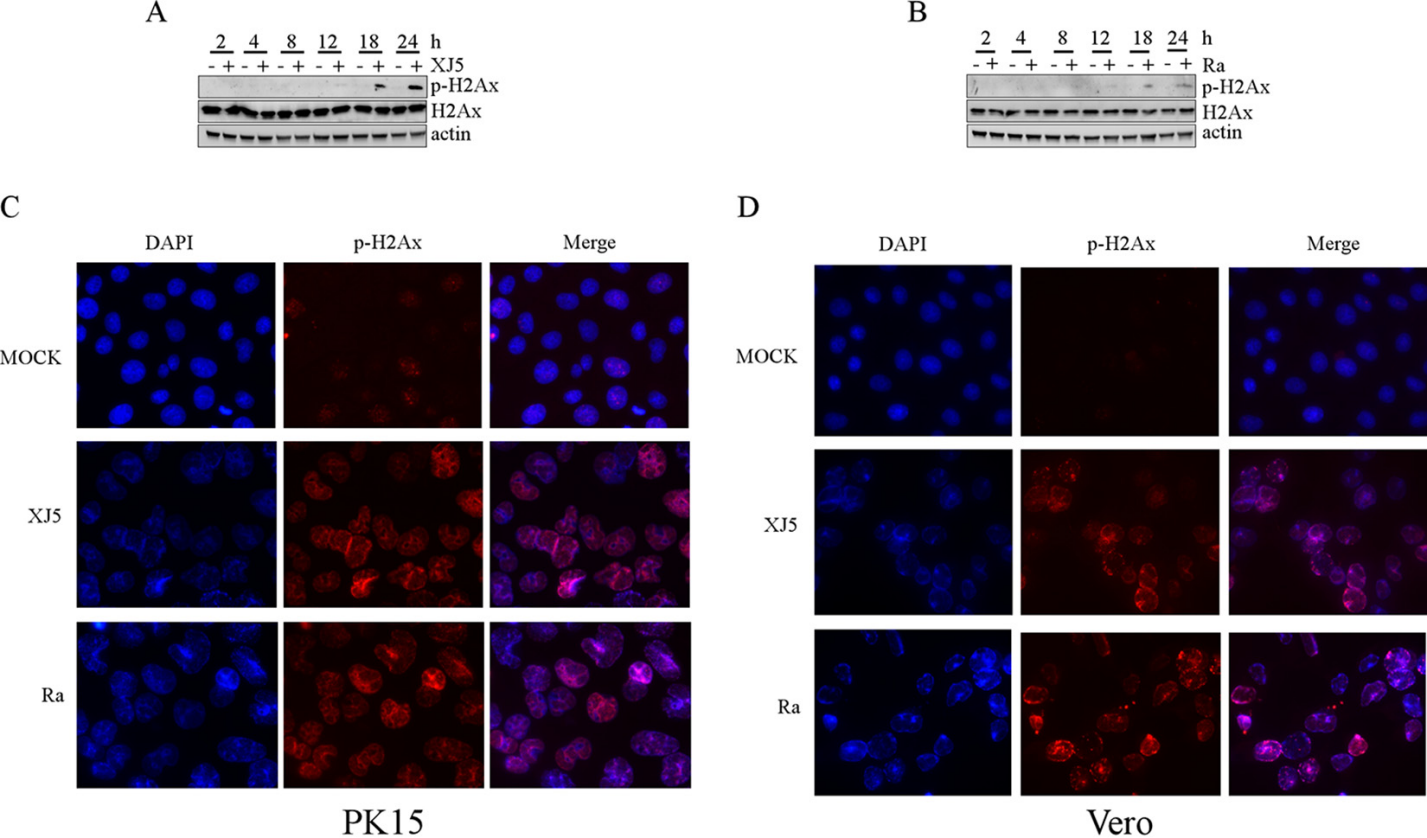
PRV replication induced the DNA damage response. (A and B) PRV XJ5 and Ra (MOI, 0.1) infected PK15 cells for 2, 4, 8, 12, 18, and 24 h. Western blotting was used to evaluate p-H2AX protein levels in PK15 cells infected with PRV XJ5 and Ra at different time points. (C and D) An immunofluorescence assay was used to detect p-H2AX in infected PK15 cells at 24 h postincubation.

H2AX is a variant of histone H2A. DNA double-strand breaks induce phosphorylation of H2AX at serine 139 to generate γ139. PRV XJ5 and Ra (MOI, 0.1) were used to infect PK15 cells for 2, 4, 8, 12, 18, and 24 h. The γ-H2AX expression was evaluated by immunoblotting. The results revealed that PRV XJ5 and Ra infection increased the expression of γ-H2AX in PK15 cells compared with that in mock-infected cells at 18 and 24 hpi (Fig. 5A and B). Furthermore, the phosphorylation of H2AX at serine 139, the most sensitive marker of DDR in PK15 and Vero cells, was detected using IFA. After 24 h of infection, the percentage of γ-H2AX-positive cells significantly increased (Fig. 5C and D), which indicated that PRV replication induced DDR.

### ATM inhibitor KU55933 decreased PRV replication.

**FIG 6:**
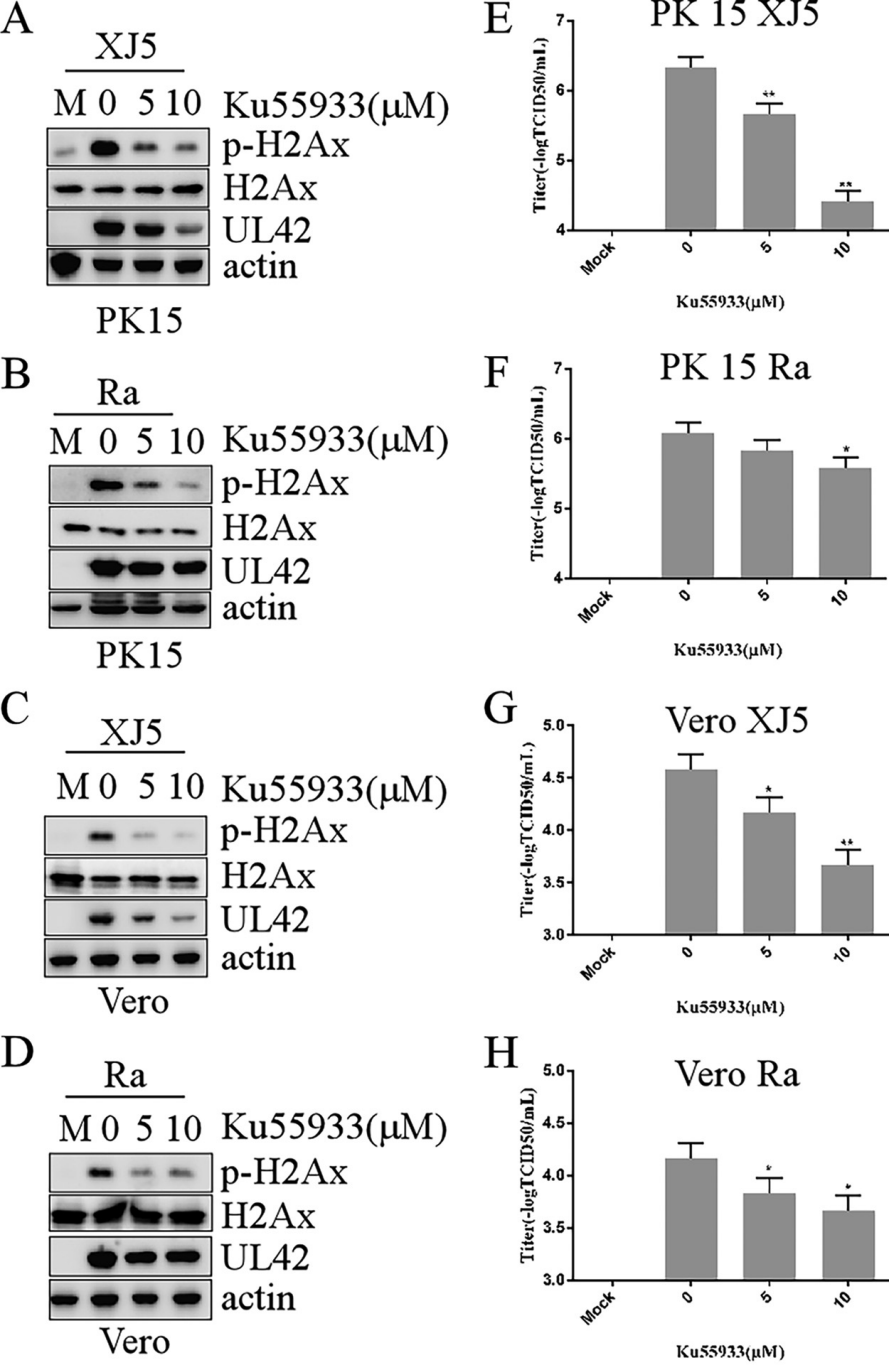
KU55933 decreased PRV replication. PK15 and Vero cells were pretreated with different concentrations of KU55933 for 1 h before being infected with PRV XJ5 or Ra (MOI, 0.1) and were then treated with different concentrations of KU55933 for 24 h. Intact cells and supernatants were collected at 24 h postincubation. (A to D) p-H2AX, UL42, and actin proteins were detected by Western blotting. (E to H) Viral titers were determined using the 50% tissue culture infectious dose assay. Values represent means ± SD from three independent experiments. *, *P* < 0.05; **, *P* < 0.01.

H2AX is a substrate of several PIKKs, such as ATM, ATR, and DNA-PKCs. We first assessed the cytotoxic effect of ATM inhibitor KU55933 for 24 h, and we found no cytotoxic effects of tubacin from 5 to 10 μM (data not shown). To investigate PIKKs effects on PRV replication, the ATM inhibitor KU55933, ATR inhibitor VE-821, and DNA-PK inhibitor NU7441 were used to pretreat PK15 and Vero cells for 1 h at 37°C, and then the cells were infected with PRV Ra and XJ5 (MOI, 0.1). At 24 hpi, the cells and supernatants were harvested for Western blotting and viral titer analysis. Figure 6A to D illustrates a marked reduction in PRV replication by KU55933. Viral titer assays indicated that KU55933 inhibited PRV replication (Fig. 6E to G). However, VE-821 and NU7441 had no effects on PRV replication in PK15 cells (data not shown). Taken together, these results showed that KU55933 reduced PRV replication, revealing that ATM-dependent DDR regulated PRV replication.

### Inhibition of HDAC6 reduced DDR.

**FIG 7:**
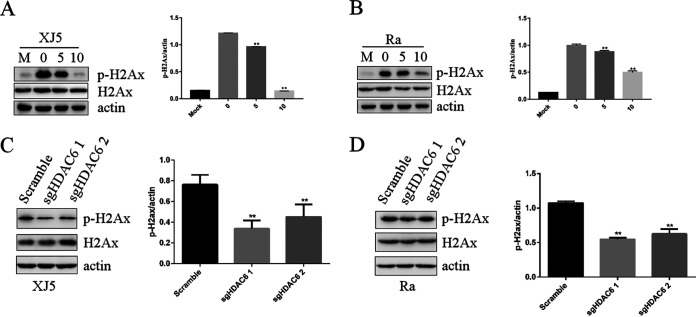
Inhibition of HDAC6 decreased the PRV-induced DNA damage response. (A and B) PK15 cells were pretreated with different concentrations of tubacin for 1 h before being infected with PRV XJ5 or Ra (MOI, 0.1) and were then treated with different concentrations of tubacin for 24 h. Intact cells were collected at 24 h postincubation. p-H2AX and actin proteins were detected by Western blotting. (C and D) PK 15 cells with *HDAC6* knocked out were infected with PRV XJ5 or Ra for 24 h. p-H2AX and actin proteins were detected by Western blotting. The relative intensity of protein was visualized by Image J. Values represent means ± SD from three independent experiments. **, *P* < 0.01.

The effects of HDAC6 on DDR in PK15 were determined. PK15 cells were pretreated with tubacin (5 and 10 μM) or control DMSO for 1 h at 37°C. The cells were infected with PRV XJ5 or Ra (MOI, 0.1) for 24 h. The Western blotting results revealed that tubacin reduced γ-H2AX protein levels (Fig. 7A and B). *HDAC6*-knockout PK15 cells were used to detect γ-H2AX expression. Western blotting results showed that the level of DDR was lower in sgHDAC6-1- and sgHDAC6-2-transfected cells than in NC-transfected PK15 cells (Fig. 7C and D). Altogether, these results indicated that HDAC6 inhibition decreased DDR.

## DISCUSSION

HDAC6 is a cytoplasmic enzyme that mediates various biological functions through its deacetylase and ubiquitin-binding activities. HDAC6 is involved in immune synapse formation (23, 24), misfolded protein degradation (25), stress response (26), and virus infection. Previous studies showed that HDAC6 is implicated in virus infection, for example, HIV-1 fusion and infection were inhibited by HDAC6-mediated acetylation of α-tubulin (21), and HDAC6 inhibited influenza A virus replication (19, 20). In this study, we first confirmed that PRV replication promoted the expression of HDAC6 in PK15 and Vero cells. Then, we demonstrated that tubacin- or sgRNA-induced HDAC6 inhibition significantly inhibited PRV replication and that overexpression of HDAC6 increased PRV replication. These results suggested that HDAC6 effectively regulated PRV replication. Thus, HDAC6 can be a novel antiviral target.

When cells receive DNA damage, cells initiate a complex signaling cascade called the DNA damage response (DDR). DDR is a cascade of phosphorylation processes which sense single- or double-strand DNA breaks. DDR regulates multiple signaling pathways, including DNA repair and apoptosis. DDR recruits DNA repair machinery to DNA damage sites and causes cell cycle arrest, which allows cells arrested in certain phases to repair the DNA damage (27). ATM, ATR, and DNA-PKCs belong to the same PIKK family. A study demonstrated that H2AX phosphorylation could be inhibited by the kinase inhibitor wortmannin (28), suggesting that H2AX can be a substrate for PIKKs. Many studies have shown that some viruses can hijack DDR to increase viral replication through different mechanisms (29–31). Our results revealed that PRV replication facilitated H2AX phosphorylation, causing DDR. Some studies have shown that DDR is commonly encountered during several DNA and a few RNA viral infections and is important for the viral life cycle, like the herpes simplex virus 1 (HSV-1), Zika virus, and Marek’s disease virus (32). This has been particularly evidenced in herpesvirus infections, for which ATM and ATR DNA damage pathway proteins play beneficial roles for viral replication (32). Alphaherpesviruses HSV-1 and HSV-2 can cause ATM-dependent DDR (33, 34). DDR is a self-protective mechanism against virus infection. After the virus infects the host cell, the cell provides the material basis for DNA replication, transcription, and RNA processing of the virus and also provides conditions for the integration of viral DNA into its chromosomes to establish lysogenic infection and regulate the expression of viral genes (35). Viral infection induces DDR through different mechanisms, and viruses have also evolved different mechanisms to counter the host cell's DDR (31, 36–38). There is a complex relationship between virus and DDR. Therefore, we explored the relation between PRV and DDR. We demonstrated that PRV infection induced H2AX phosphorylation, and KU55933 significantly inhibited H2AX phosphorylation and PRV infection *in vitro*. But the ATR inhibitor VE-821 and the DNA-PK inhibitor NU7441 had no effect on PRV infection. The results revealed that PRV induced an ATM-dependent DNA damage response to promote its replication. Our results are consistent with previous reports (39).

HDAC6 is now considered to be a master regulator of the cellular response to cytotoxic assaults (26, 40, 41) and plays a role in genotoxic stress responses (14, 42, 43). HDAC functions are protective against DNA damage in cancer cells. The HDAC 6/8/10 inhibitor TH34 can cause DNA damage-mediated cell death in human high-grade neuroblastoma cell lines (44). Knockdown of HDAC6 enhances cisplatin-induced DNA damage in non-small cell lung cancer cells (43). HDAC6 is a guardian of irradiation-induced DNA damage and stemness in glioblastoma (45). The HDAC6 inhibitor tubacin enhances DNA damage induced by etoposide or suberoylanilide hydroxamic acid (SAHA) in transformed cells (42). However, the relation between HDAC6 and DDR has not been describe for PRV infection. Therefore, we utilized the HDAC6 inhibitor tubacin, HDAC6 sgRNA, and the overexpression of HDAC6 to check the effect on DNA damage. Our research revealed that tubacin- or sgRNA-induced HDAC6 inhibition decreased DNA damage induced by PRV replication and the overexpression of HDAC6-induced DNA damage. Thus, HDAC6 regulated DNA damage to affect PRV replication. Some studies revealed that HDAC6 regulated DDR and mismatch repair activities through maintaining MutS homeostasis via deacetylation and ubiquitylation of MSH2 (14), and HDAC6 regulated DDR by disrupting the assembly of the MutS-MutL complex via deacetylation of MLH1 (46). But how HDAC6 regulates DDR during PRV infection will need to be further clarified.

In conclusion, when PRV infects cells, PRV promotes the expression of HDAC6, which in turn upregulates PRV replication. The data presented here strengthen the claim that HDAC6 is an important target to trigger PRV replication. Furthermore, we showed that HDAC6 induced DDR, and the DDR inhibitor KU55933 decreased PRV replication. These results suggested a novel mechanism by which HDAC6 regulates DDR to promote PRV replication.

## MATERIALS AND METHODS

### Cells and virus.

PK15, Vero, and HEK293T cells were cultured in Dulbecco’s modified Eagle’s medium (Sigma, USA) supplemented with 5%, 6%, and 10% fetal bovine serum (Lonsa), respectively, at 37°C in 5% CO_2_. PRV wild-type (PRV Ra) and variant strains (PRV XJ5) were used for infection, as previously described (47).

### Reagents and antibodies.

Tubacin was purchased from GlpBio (catalog number GC16386), and 10 mM stock solutions were prepared with DMSO and stored at −20°C for all subsequent experiments. KU55933 was purchased from Selleck (catalog number S1092), and 10 mM stock solutions were prepared with DMSO and stored at −20°C for all subsequent experiments.

HDAC6 and phosphorylated H2AX (p-H2AX; Ser139; γ-H2AX) were obtained from Proteintech (catalog number 12834–1-Ag) and CST (catalog number 9718S). UL42, gE, PRV-positive serum, β-actin, fluorescein isothiocyanate (FITC)-conjugated goat anti-pig IgG antibody, and 4',6-diamidino-2-phenylindole were used as previously described (47).

### Overexpression of *HDAC6* in Vero cells.

*HDAC6* was amplified from the cDNA of Vero cells and then cloned into the vector pcDNA4. The nucleotide sequences of the plasmids expressing *HDAC6* were determined to ensure that the correct clones were used in this study. Vero cells were seeded in 6-well plates and cultured for about 20 h. After reaching 60% confluence, cells were transduced with 4 μg/dish pcDNA4 or pcDNA4-HDAC6 using FuGENE. At 24 h postincubation, the cells were infected with PRV Ra or XJ5. The cells and supernatants were harvested for further analysis at 24 hpi.

### *HDAC6* knockout using the clustered regularly interspaced short palindromic repeats system.

Human HEK293T cells were seeded in 6-well plates and cultured for about 20 h. After reaching 60% confluence, the cells were transfected with 4 μg plasmids, including sgRNA plasmid (primers are shown in Table 1), pLp1 plasmid, pLp2 plasmid, and pLp VSVG plasmid by using FuGENE. At 36, 48, and 72 hpi, the culture media containing the viruses were collected, filtered through a 0.45-μm membrane, and stored at −80°C. For cell infection, PK15 cells were cultured in the 6-well plates. After reaching 80% confluence, the cells were infected with 1 mL lentivirus-containing medium mixed with 1 mL fresh medium. After 48 h, the medium was changed and the cells were cultured for a further 48 h. The cells were screened in the culture medium containing puromycin (2 μg/mL) and incubated for about 48 h. The control cells died. Additionally, a qPCR was performed to detect and confirm the positive lentivirus infection. Finally, PRV Ra and XJ5 infections were performed at an MOI of 0.1 each in the positive cells.

**TABLE 1:** sgRNAs targeting porcine *HDAC6*

Name	Primer sequence
sgHDAC6-F1	CACCGAGGTGACGCTGGAGTCGTGT
sgHDAC6-R1	AAACACACGACTCCAGCGTCACCTC
sgHDAC6-F2	CACCGAAATTGGCGGCGCGCGCCA
sgHDAC6-R2	AAACTGGCGCGCGCCGCCAATTTC

### Pharmacological inhibitors.

The HDAC6 inhibitor tubacin and the p-H2AX inhibitor KU55933 were used at 5 μM and 10 μM, respectively. PK15 and Vero cells were pretreated with the inhibitors or the control DMSO for 1 h at 37°C. Then, PK15 and Vero cells were infected with PRV Ra and XJ5 (MOI, 0.1), respectively. After 24 hpi, the cells and supernatants were harvested for further analysis.

### qPCR.

Total RNA was isolated using TRIzol reagent (Thermo) and was subjected to cDNA synthesis using the HiScript III RT SuperMix for qPCR (+gDNA wiper; Vazyme, China). The qPCR was performed in triplicate using ChamQ Universal SYBR qPCR master mix (Vazyme, China), and data were normalized based on the β-actin expression of each sample. The threshold cycle (2^−ΔΔ^*^CT^*) method was used to calculate relative expression changes. Primers used for qPCR are presented in Table 2.

**TABLE 2:** Primers of *HDAC6* for reverse transcrition-qPCR

Name	Primer sequence
HDAC6-F	ACTCCAGCGTCACCTCGAAG
HDAC6-R	GCCCCACGATTAGGTCTTGCTC

### Virus titration.

The virus titer assays were performed as previously described (47).

### Western blotting.

The Western blotting procedure was performed as previously described (47). PRV gE, UL42, HDAC6, p-H2AX, and actin proteins were detected using the respective primary and secondary antibodies.

### IFA.

IFA was performed as previously described (21). Briefly, PK15 and Vero cells were inoculated with PRV Ra and XJ5 (MOI, 0.1), respectively. At 24 hpi, the cells were fixed with 4% paraformaldehyde, permeabilized with 0.1% TritonX-100, blocked with 3% bovine serum albumin, and incubated with anti-PRV-positive serum, anti-p-H2AX antibody, FITC-conjugated goat anti-pig IgG antibody, and Alexa Fluor 555-conjugated goat anti-rabbit secondary antibody (Invitrogen, USA). All images were taken at 40× magnification.

### Statistical analysis.

Values are presented as means ± standard deviations (SD). Data were analyzed using Student's *t* test and Prism 5. *P* values of <0.05 were considered significant (*, *P* < 0.05; **, *P* < 0.01).
